# Laminar firing and membrane dynamics in four visual areas exposed to two objects moving to occlusion

**DOI:** 10.3389/fnsys.2013.00023

**Published:** 2013-06-25

**Authors:** M. A. Harvey, P. E. Roland

**Affiliations:** ^1^Laboratory of Brain Research, Department of Neuroscience, Karolinska InstituteSolna, Sweden; ^2^Department of Neuroscience and Pharmacology, University of CopenhagenCopenhagen, Denmark

**Keywords:** feedback, lateral interactions, visual cortex, visual motion, voltage sensitive dye

## Abstract

It is not known how visual cortical neurons react to several moving objects and how their firing to the motion of one object is affected by neurons firing to another moving object. Here we combine imaging of voltage sensitive dye (VSD) signals, reflecting the population membrane potential from ferret visual areas 17, 18, 19, and 21, with laminar recordings of multiunit activity, (MUA), when two bars moved toward each other in the visual field, occluded one another, and continued on in opposite directions. Two zones of peak MUA, mapping the bars' motion, moved toward each other along the area 17/18 border, which in the ferret maps the vertical meridian of the field of view. This was reflected also in the VSD signal, at both the 17/18 border as well as at the 19/21 border with a short delay. After some 125 ms at the area 19/21 border, the VSD signal increased and became elongated in the direction of motion in front of both of the moving representations. This was directly followed by the phase of the signal reversing and travelling back from the 19/21 border toward the 17/18 border, seemingly without respect for retinotopic boundaries, where it arrived at 150 ms after stimulus onset. At this point the VSD signal in front of the moving bar representations along the 17/18 border also increased and became elongated in the direction of object motion; the signal now being the linear sum of what has been observed in response to single moving bars. When the neuronal populations representing the bars were some 600 μm apart on the cortex, the dye signal and laminar MUA decreased strongly, with the MUA scaling to that of a single bar during occlusion. Despite a short rebound of the dye signal and MUA, the MUA after the occlusion was significantly depressed. The interactions between the neuronal populations mapping the bars' position, and the neurons in between these populations were, apart from 19/21 to 17/18 interaction, mainly lateral-horizontal; first excitatory and inducing firing at the site of future occlusion, then inhibitory just prior to occlusion. After occlusion the neurons that had fired already to the first bar showed delayed and prolonged inhibition in response to the second bar. Thus, the interactions that were particular to the occlusion condition in these experiments were local and inhibitory at short cortical range, and delayed and inhibitory after the occlusion when the bars moved further apart.

When an object is moving into the visual field of view and the retina is still, the object is mapped in several visual areas as moving peak firing rates (Motter and Mountcastle, 1981; Harvey et al., 2009). This mapping is associated also with moving increases in the population membrane potential (Jancke et al., 2004; Yang et al., 2007; Harvey et al., 2009). Increases in the population membrane potential reflect the local dynamics of the neurons, but also dynamics influenced by activity in higher visual areas, (Harvey et al., 2009). Not only feed-forward input, but also lateral horizontal neuronal computations and action potentials from higher order areas influence visual perception and firing rates in the primary visual cortex (Gilbert and Wiesel, 1989; Lamme, 1995; Bosking et al., 1997; Bringuier et al., 1999; Buzas et al., 2006; Roland et al., 2006; Roland, 2010). The relative weights of these three inputs are still debated. Feed-forward input from the lateral geniculate nucleus affects the computations of neurons primarily located in layer 4 of primary visual cortex, (Maunsell and Gibson, 1992; Hirsch et al., 2002). Horizontal interactions within an area may take place in supra- and infra-granular layers, and these layers are also assumed to be the target of back projecting axons from higher areas. It is known that another object outside a receptive field occupied by an object can influence the spiking from that receptive field (Jones, 1970; Bishop et al., 1973; Allman et al., 1985). The other object can facilitate or inhibit the spiking from the receptive field, depending on its distance from the field (Jones, 1970; Bishop et al., 1973). The current picture is that long-range horizontal connections are excitatory for similar stimulus orientations whereas short-range axons have no orientation preference and are mainly inhibitory, (Tucker and Fitzpatrick, 2003). Visually moving objects add complexity to this picture. A moving object, as a stationary object, elicits a laterally spreading excitation in the supragranular layers, but this is soon superimposed upon by excitation and firing ahead of the object mapping in the direction of object motion, (Harvey et al., 2009).

When a single object moves in the field of view and the eyes don't move, the object is mapped in a retinotopic fashion in visual area 17 as increased multiunit activity (MUA) and increased membrane potential by one population of neurons (Jancke et al., 2004; Harvey et al., 2009). This population forms a path in the cortex, corresponding to the trajectory in the field of view. For each position of the object in the field of view, there is one sector of this population where the object is mapped as the peak of the MUA and membrane potential increase (Harvey et al., 2009). However, after some 130 ms, the neurons not yet having mapped the object, start to fire and increase their membrane potential as far as 8° ahead, thus marking the future trajectory of the peak activity across cortex. (Harvey et al., 2009). The present study is an extension of the Harvey et al. (2009) study with the purpose of revealing the cortical dynamics elicited by two bars moving in opposite directions.

When more than one object traverses the visual field simultaneously, neurons firing to one object might be affected by other neurons firing to another object in a way that is not possible to predict from the dynamics associated with a single moving object. This happens for example when two objects move to occlude or partially occlude one another (Adelson and Movshon, 1982). This situation is common in natural scenes. A moving object is mapped by spatially extended populations of neurons in all layers of the visual cortex (Harvey et al., 2009), but to our knowledge the spatio-temporal representations and interactions between two continuously moving objects has not been examined neurophysiologically. We used a simplified visual scene consisting of a gray background and two objects, white bars, moving in opposite directions to occlude one another (Figure 1). We examined the relative changes in population membrane potential in supragranular layers of areas 17, 18, 19, and 21 with voltage sensitive dyes (VSDs), as well as the laminar MUA in areas 17 and 18 of ferrets.

**Figure 1:**
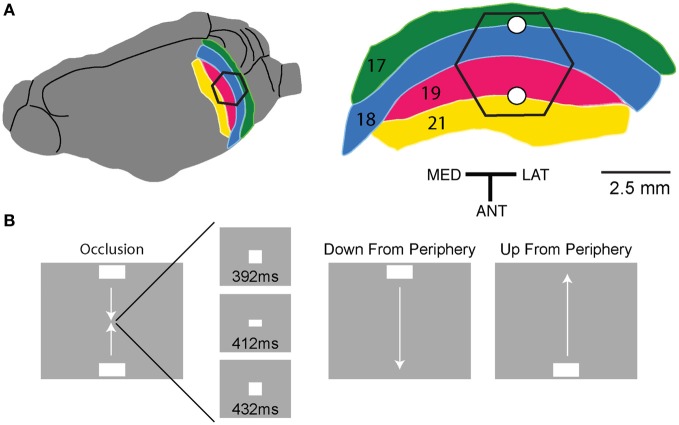
**Experimental conditions and ferret visual areas. (A)** The left hemisphere of the ferret brain with visual areas 17, 18, 19, and 21. The cortex monitored by the hexagonal photodiode array is delimited in black. The cytoarchitectural borders between areas 17 and 18 and between areas 19 and 21 correspond to the mapping of the vertical meridian in the field of view on the cortex. The two white dots mark the expected mappings of the center of field of view. The relation between hexagon borders and cytoarchitectural borders (and hence the center of field of view mapping) varies somewhat between animals. Each diode picks up a signal from a cortical spot of 150 μm in diameter. MED, medial; LAT, lateral; and ANT, anterior direction. **(B)** The stimulus conditions and timing of the occlusion. All stimulus conditions were compared to a gray screen (baseline condition). All latencies relate to the appearance of the stimuli on the display screen.

From the single bar dynamics, we predicted that four populations of neurons would map the two bars in the cortex, i.e., two representations, one of each bar, at the 17/18 border and two other representations at the 19/21 border. Based on previous work (Harvey et al., 2009), we expected to see evidence for communications between areas 19/21 and 17/18. Finally based on Harvey et al. (2009) we expected an increase in membrane voltage ahead of the representations along the future cortical trajectory of object motion. Our results confirmed these predictions, and open the possibility that populations of neurons could provide an advanced signal of the location of an upcoming occlusion.

We also tested the hypotheses that inter-area and cross-area interactions would be non-linear. To our surprise we found both linear and non-linear interactions. The dynamics of the cortical interactions prior to occlusion depended on the distance between the objects, but after the occlusion they were largely independent of the distance between objects. The interactions started early at a long range and initially they were net excitatory. Close to occlusion the population membrane potential showed strong net decreases of excitation, or alternatively net increases in inhibition. After the occlusion, the previous mapping of the objects continued to affect the MUA.

## Materials and methods

### Animals

All experimental procedures were approved by the Stockholm Regional Ethics Committee and were performed according to European Community guidelines for the care and use of animals in scientific experiments. Recordings were performed in 14 adult, female ferrets. Ferrets were initially anesthetized with Ketamin (15 mg kg^−1^) and Medetomidine (0.3 mg kg^−1^) supplemented with Atropine (0.15 mg kg^−1^). After the initial anesthesia ferrets received a tracheotomy and were ventilated with 1:1 N_2_0:0_2_ and 1% Isoflurane. The arterial pCO_2_ (partial pressure of CO_2_) was maintained between 3.5 and 4.3 KPa. A craniotomy was made exposing the left hemisphere visual areas 17, 18, 19, and 21 and was covered with a chamber affixed to the skull with dental acrylic. Animals were paralyzed with pancuronium bromide (0.6 mg kg^−1^), the left eye was occluded, and the right eye had its pupil dilated (1% atropine), nictating membrane retracted (10% Phenylephrine), and was then fitted with a zero power contact lens.

### Stimulation and imaging

A reverse ophthalmoscope was used to record the position of the optic disk and center a video monitor to the area centralis. Known cortical landmarks were then used to guide a single electrode penetration at the estimated crossing of the vertical and horizontal meridian. The receptive field (RF) at this area was then mapped using an m-sequence method, (Reid et al., 1997). The monitor position was then further adjusted so as to be precisely centered to this RF location. The cortex was stained for 2 h with the VSD RH1838 (0.53 mg ml^−1^; *n* = 3) or RH1691 (0.53 mg ml^−1^; *n* = 11) (Optical Imaging, Rehovot, Israel). After staining, the cortex was rinsed with artificial cerebral spinal fluid, the chamber was filled with silicon oil and sealed with a cover glass. Imaging was centered on the initial recording site and acquired using a 464-channel photodiode array, (H469-IV WuTech Instruments Gaithersburg, MD) through a macroscope fitted with a 5× objective (Red Shirt New Haven, CT). Images were acquired at a rate of 1.6 kHz, stimulus presentation was synchronized to the ECG signal, and respiration stopped during stimulus presentation. Stimuli were presented in a pseudorandom order on a video monitor with a refresh rate of 120 Hz located 57 cm in front of the animal. Stimuli were controlled using a VSG series IV system (Cambridge Research Systems, Kent UK). Stimuli consisted of 1 × 2° horizontal bars (64.5 cd m^−2^) on a homogenous gray background (7.2 cd m^−2^). There were three stimulus conditions: (1) upward and (2) downward moving bars *originating* 10.5° below and 10.5° above the center of field of view (CFOV), respectively, and moving a total of 21° with a velocity of 25.4° s^−1^ for a period of 825 ms with start and end points equidistant from the screens center. (3) In the occlusion condition, upward and downward moving bars were presented simultaneously 10.5° below and 10.5° above the CFOV moving toward each other with a velocity of 25.4° s^−1^ for a period of 392 ms. At 392 ms the bars abutted one another, such that for a short moment, 8 ms, they occupied 2 × 2° square in the CFOV (Figure 1). Then the bars began to occlude one another until there was, at 412 ms, only the image of a single bar at the center of the screen. From 412 to 432 ms this central bar grew until again the bars were at an abutting position. From 432 ms until 825 ms the bars moved away from each other until they reached their final positions *10.5° below and 10.5° above the CFOV* at which point they disappeared.

### Electrophysiology

Electrode penetrations were made perpendicular to the cortical surface along the estimated course of the vertical meridian using single shank, 16 channel, laminar probes (NeuroNexus, Ann Arbor, MI) with recording site resistances of ~3 MΩ, and separated by 100 μm. Signals were routed through an RA16AC head stage to an RA16PA Medusa preamplifier and amplified at 40 K using the RA16 Medusa Base station (Tucker-Davis Technologies, Alachua, FL). For multiunit recordings the signal was digitally band pass filtered between 100 Hz–10 KHz and for local field potential recordings between 1 and 10 KHz. Signals were acquired and written to a hard-drive using CED power 1401 AD-converter and Spike 2 Software (Cambridge Research Systems). All subsequent analysis was done using Matlab R13 (The MathWorks, Natick, MA). At each recording site receptive fields were first mapped using the methodology noted above.

### Cytorarchitectonics and functional retinotopy

At the end of the experiment three vertical needle marks were made around the recorded area, the animals were sacrificed (pentobarbital) and perfused transcardially with 4% paraformaldehyde. Brains were sectioned and alternate 50 μm sections were stained for Nissl and cytochrome oxidase. Areal borders were then reconstructed using cytoarchitectonic landmarks (Innocenti et al., 2002), and these borders were mapped onto the image of the cortical surface in each animal. After reconstruction, the cytoarchitectural borders of individual animals were aligned by simultaneous standard affine transformations as described in Harvey et al. (2009) (see Movies S1, S3).

**Figure A1:**
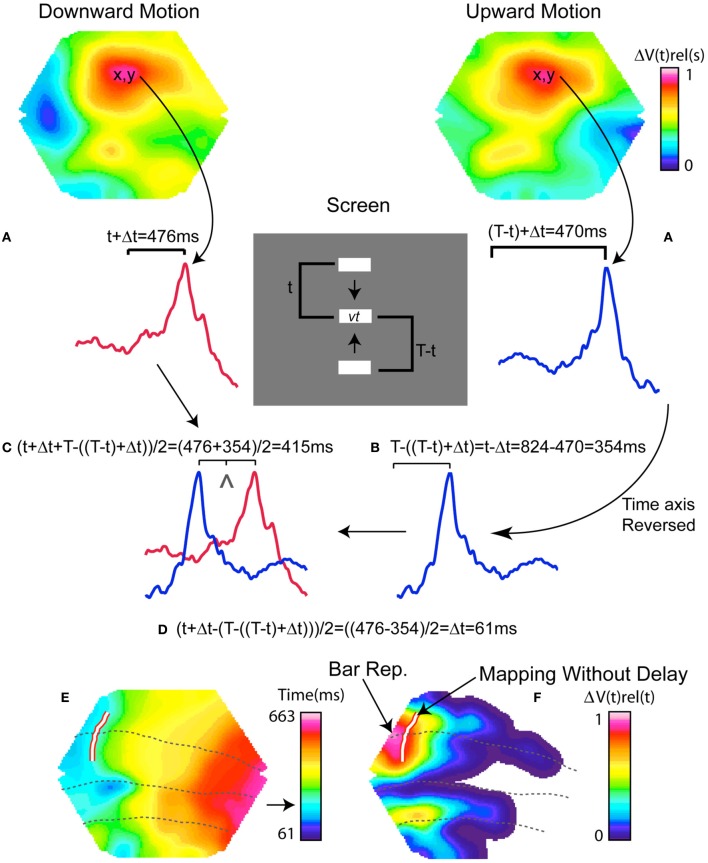
**Mapping of the mean maximum Δ*V*(*t*) without delay: in order to get an estimate of the position of the object representation on the cortex at any given time, we used the strategy of Kalatsky and Stryker (2003)**. Briefly, the contrast bar moves with a velocity *v* over the screen, starting at 0 ms at the top/bottom of the screen and ending at *T* ms at the bottom/top of the screen, here *T* = 824 ms. After a certain time, *t*, the bar position on the screen, *vt*, is mapped to a cortical position (*x, y*). The Δ*V*(*t*) at position (*x, y*) reaches its maximum after a certain delay, Δ*t*, after the bar has arrived at screen position *vt*. Thus, the maximal Δ*V*(*t*) will be reached after *p*^+^ = *t* + Δ*t*, (**A** Left). When the bar moves in the opposite direction, the square will reach the same cortical position (*x, y*) after (*T* − *t*) and hence the maximal Δ*V*(*t*) will be reached after *p*^−^ = (*T* − *t*) + Δ*t* ms, (**A** Right). The color scale at the right shows the Δ*V*(*t*)rel values and hence also the peak value which is reached at 476 ms for the downward motion condition and at 470 ms for the upward motion condition in this animal. The time courses of the respective Δ*V*(*t*)'s for the cortical point *x, y* are shown below the snapshots. If one reverses the time axis for one of the conditions one will end up with the same stimulus as the other condition, i.e., *T* − (*T* − *t*) = *t*. However, the maximal response will instead come Δ*t before* the arrival time of the stimulus at point vt on the monitor, **(B)**. Now the time when the stimulus passes the position vt on the monitor can be found by averaging *p*^+^ and *T* − *p*, which in this case equals the position (*x, y*) on the cortex without any delay, **(C)**. We can now calculate the delay of the maximal response Δ*t*, by subtracting *p*^+^ and *T* − *p*, The time that the peak would have if there were no delay from the screen to the cortex is calculated to be 415 ms after the start of the motion of the bars = start of the bar stimuli **(D)**. Once we have the value derived in **(C)** for *every* imaged point, (*x, y*), we can construct a map of the bar position without delay as shown in **(E)**. Note that the point *x, y* used in the example above is not marked in **(E)**. Instead the position of the Δ*V*(*t*) maximum is mapped as a white line, exactly at the time when the excitation ahead of the bar mapping, the SRP, was maximal. Note that the colors now show time from the start of stimulation. The arrow shows the time when the Δ*V*(*t*) at the white line was maximal. **(F)** For comparison, a snapshot of the Bonferroni corrected Δ*V*(*t*) map is shown at a corresponding time point [i.e., when the (predictive) spatially restricted pre-depolarization (SRP) is maximally ahead of the bar mapping during *downwards* motion, and where the bar map would have been (white line) if there had been no delay from the display screen to the cortex].

Although recordings of the optical intrinsic signal in many cases is helpful in showing the border between areas 17 and 18, this method has not consistently shown the borders between area 18 and 19, nor the borders between area 19 and 21 or between area 21 and the suprasylvian area in the ferret. We used the VSD signal, Δ*V*(*t*), to determine the location of the stimulus-induced peak of Δ*V*(*t*) in all four areas according to the method devised by Kalatsky and Stryker (2003). In the control conditions the bar moved upwards in half the trials and downwards in the other half. Thus, the bar would reach an identical position on the screen from two different directions. As each position on the screen corresponds to one cortical position along the 17/18 border, we could estimate how the bar would be mapped on the cortex if there was no delay between its position on the screen and its mapping as the Δ*V*(*t*) peak on the cortex (Kalatsky and Stryker, 2003). Figure A1 illustrates this procedure and Movie S1 panel **C** shows the locations of our peak Δ*V*(*t*) estimate. This map, color coded in Figure A1, then serves as an additional independent mapping of the retinotopy obtained from a bar moving along the vertical meridian. The velocity of the moving representation over the cortex predicted from this mapping without delay was 25.43° s^−1^, (s.e.m. 1.1° s^−1^, *n* = 4), which might serve as a further validation of the recording procedures. It is apparent that the bar mappings in visual areas 17, 18, 19, and 21 always lag the position of the object mapped without delay. This non-delayed representation was on average 50 ms (s.e.m. = 4 ms; *n* = 4) ahead of the maximal Δ*V*(*t*) and the maximal MUA firing rate. Note that this procedure uses information from all imaged cortical points. For further details see Figure A1.

### Data analysis

#### Treatment of the VSD signal

All VSD signals were analyzed in terms of fractional fluorescence, the details of which have been described elsewhere (Roland et al., 2006; Eriksson et al., 2008). In brief, the signal in the blank (background alone) condition is subtracted from the signal of the stimulus conditions and divided by the background fluorescence to yield the fractional fluorescence (Δ*F/F*_0_) referred to here as Δ*V*(*t*).

Two types of normalization procedures were used, normalization to the maximum Δ*V*(*t*) value in time, Δ*V*(*t*)rel(*t*) = Δ*V*(*t*)/max_*t*_ [Δ*V*(*t*)] and normalization the maximum Δ*V*(*t*) value in space, Δ*V*(*t*)rel(*s*) = Δ*V*(*x, y, t*)/max_*x, y*_ [Δ*V*(*x, y, t*)]. For the spatial normalization this meant that for each frame of our VSD measurement (0.616 ms) the Δ*V*(*t*) from the photodiode with the highest value would be set to 1 and the Δ*V*(*t*) from all other diodes would be relative to that. Finally a specific additional normalization scheme was used. In this procedure the Δ*V*(*t*) from each diode is made relative to itself within a 25 ms sliding window, such that for each time point Δ*V*(*t*)rel = Δ*V*(*x, y, t*)/max_*t*{−12.5,12.5}_[Δ*V*(*x, y, t*)]. Using this scheme we can then monitor when the Δ*V*(*t*) at each diode reaches its maximum relative to its self, rather than relative to the Δ*V*(*t*) of the surrounding diodes.

**Figure A2:**
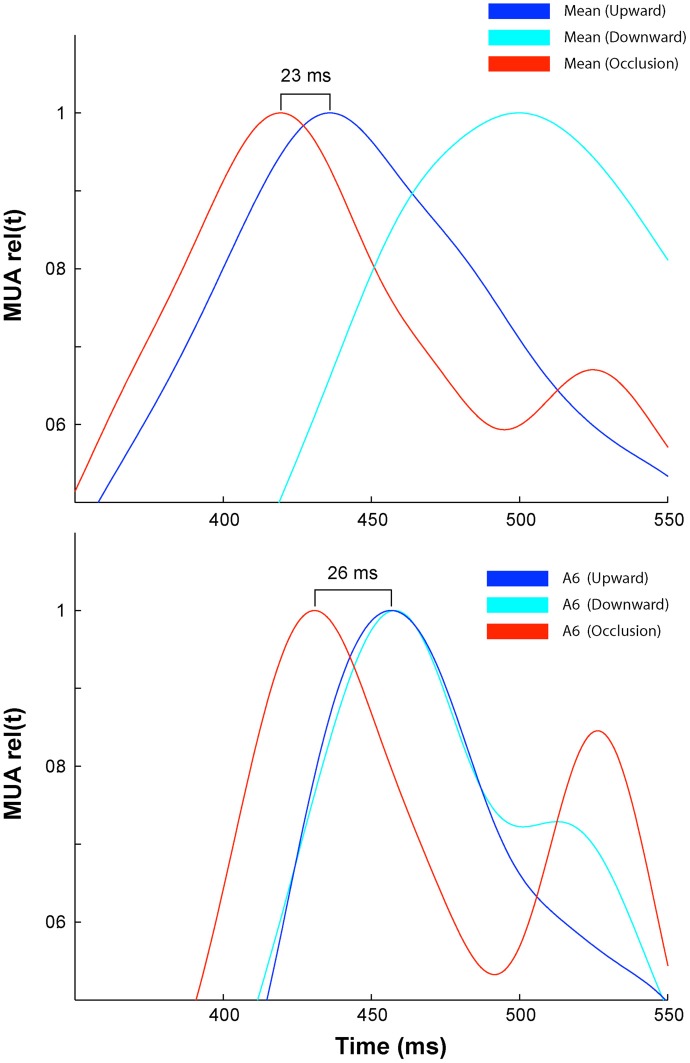
**Comparison of the mean, (*N* = 10 Animals), MUA to that obtained from a single animal, (A6) that showed identical peak times in response to upward and downward moving bars at the cortical site representing the central field of view**. Note that in both cases the peak of the response arrives equivalently earlier in the occlusion condition. MUAs are normalized in time i.e., the maximum MUA in the post stimulus interval is set to 1, in order to visualize the relative timing of the peak activity generated during the three stimulus conditions.

We selected five sites along the 17/18 cytoarchitectural border and hence the path of the moving Δ*V*(*t*) maximum at the 17/18 border, and three sites along the 19/21 cytoarchitectural border and the path of the moving Δ*V*(*t*) maximum at the 19/21 border for detailed analysis. The purpose was to examine whether the Δ*V*(*t*) and MUA added linearly until the time of occlusion, with some caveats. First we have no systematic multiunit recordings at the 19/21 border. Second, while the distance from the site mapping the CFOV could always be calculated precisely for the Δ*V*(*t*), the location of the individual electrode penetrations were subject to some variability. This is obvious in the average MUA recorded at the central location (Site 3 in Figure 3 MUA), where the average time of arrival of the peaks in the downwards and upwards movement conditions are offset by some 72 ms. This would correspond to an average lateral displacement of our penetrations at this site of 180 μm. Thus, in order to test whether the occlusion condition generated a larger amplitude of Δ*V*(*t*) and MUA than that for the single moving bar conditions we compared the Δ*V*(*t*) and MUA only to the larger of the two amplitudes generated by the control conditions for each temporal bin. Similarly when testing if the occlusion condition generated a weaker response than that for the control conditions, we compared it to the lesser of the two amplitudes of the control conditions for each bin. When comparing response onset and peak times, we always chose for statistical comparison the control condition that had the earliest onset or the earliest peak, respectively. As the positional error of the electrodes at the center was lateral, this meant that our comparisons at the center are biased toward the downward movement condition. However, data from individual animals for which there was no such discrepancy in the electrode positions do not contradict our main results as shown in Figure A2. Finally, primarily due to differences in the cortical vasculature, it was rare for two electrode penetrations in different animals to be at exactly the same distance from the cortical site mapping the CFOV, therefore the distances from center for the MUA are described as mean values in Figure 3.

#### Calculating significance

Using the amplitude fluctuations in the pre-stimulus interval to define the noise level, the Δ*V*(*t*) was thresholded at *p* < 0.01 of being noise. In this we assumed the amplitude fluctuations to be not significantly different from a Gaussian distribution. This significance threshold applied for single photodiode channels and small regions of interest consisting of three channels (see below and Figures 3–6). Once statistically significant epochs of Δ*V*(*t*) changes were determined for a particular region of interest, the timing of the first significant frame in the first post-stimulus epoch > 10 ms was the onset latency, thus calculated from the start of the stimulus. The peak latency was simply the mean peak time calculated across animals from stimulus onset (in a statistically significant epoch).

Statistics for the whole photodiode array of 464 channels. A threshold of estimated *p* < 0.01 was set for each photodiode detector channel and divided by the number of channels (464) to give the Bonferroni corrected value of *p* < 0.01.

Statistical comparisons between conditions are corrected for mass significance with a false discovery rate of 0.01 (Benjamini and Hochberg, 1995). For the movies the pre-stimulus Δ*V*(*t*) or d[Δ*V*(*t*)]/dt was thresholded with a global *p* < 0.025 or *p* < 0.01; this threshold was used on the post-stimulus Δ*V*(*t*) or d[Δ*V*(*t*)]/dt. The results are movies and snapshot sequences of only statistically significant membrane events.

In order to calculate significant responding in the MUA, a Poisson distribution was fitted to the spike trains in the pre-stimulus period *and* spikes from the background trial. Spike trains passing both the criterion of having significantly increased discharge rate compared to the pre-stimulus period of *p* < 0.01 *and* increased rate compared to the background condition of *p* < 0.01, were considered statistically significant periods of firing.

Once statistically significant epochs of MUA were determined for a particular region of interest, for the number of animals in which the region was exposed, the timing of the first frame in the first post-stimulus significant epoch > 10 ms was the onset latency, thus calculated from the start of the stimulus. The peak latency was simply the mean peak time calculated across animals from the stimulus start (in a statistically significant epoch).

## Results

VSDs are potentiometric dyes that bind non-specifically to all cell membranes, (Grinvald and Hildesheim, 2004). Changes in the fluorescence of these dyes has been shown to bear a near linear relationship to changes in membrane voltage, recorded intracellularly *in vivo* from cells in superficial cortical layers, (Petersen et al., 2003; Ferezou et al., 2006; Berger et al., 2007), as well as *in vitro*, (Cohen et al., 1974; Ross et al., 1977). We used VSDs in order to image the spatiotemporal evolution of the relative population membrane potential in the supragranular layers of ferret visual areas 17–19 and 21, when ferrets viewed two identical luminance bars moving toward each other along the vertical meridian of the field of view. In the ferret, the vertical meridian of the field of view is represented along both the cytoarchitectonic border separating visual areas 17 and 18, and along the border separating areas 19 and 21, (Manger et al., 2002), (Figure 1). Subsequently we used the results from the VSD imaging to guide the placement of a laminar electrode along the 17/18 border, and thus along the predicted path of activity evoked by the moving bars.

The VSD signal, Δ*V*(*t*), is the difference between the fluorescence recorded during a stimulus condition and that recorded during the baseline, gray screen, condition divided by the fluorescence obtained in darkness (Materials and Methods). According to a recent estimate, approximately 90–95% of this difference signal reflects the difference in synaptic activity (Berger et al., 2007). The spatio-temporal dynamics of the Δ*V*(*t*) and the MUA associated with the motion of a single bar is relatively complex. For this reason, we shortly summarize the results of the control conditions [for further details see Harvey et al. (2009)].

### Spatio-temporal dynamics of the membrane potential and multiunit activity changes associated with the motion of a single bar

**Figure 2:**
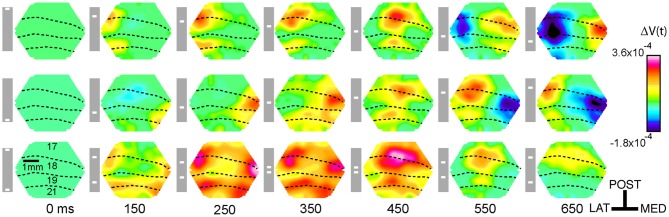
**Snapshots of the dye signal changes taken from the left hemisphere in one animal, for the three conditions (shown in the gray boxes)**. All Δ*V*(*t*) increases at yellow scale and above were significant (*p* < 0.01). As the photodiode array creates a mirror image of the cortex, lateral (LAT) is to the left and medial (MED) is to the right and posterior (POST) is up. Dashed lines show the cytoarchitectural borders of the animal. Time of the snapshots and bar position at the display screen shown in ms below each column. Note that the bar mapping on the cortex only corresponds to the maximal Δ*V*(*t*) increase in the snapshot. For the occlusion condition (last row), the Δ*V*(*t*) increase emerges in the cortex between the representation sites of the two bars at the 19/21 border, this is the slender spatially restricted pre-depolarization, SRP and the beginning of a similar SRP is seen along the 17/18 border (150 ms). After 450 ms the amplitude of the Δ*V*(*t*) decreases. Notably, we did not perform specific subtractions of the Δ*V*(*t*) from bars with other orientations, and consequently no orientation specific domains can be seen in the figures or movies as the orientation specific part of the Δ*V*(*t*) amounts to maximally 15% (Sharon et al., 2007). Data filtered with a σ = 20 frame temporal filter. The snapshots cannot reveal the full dynamics, for this the reader should look at the Movies S1–S3.

There were two control conditions, (Figure 1B), in which a single bar moved upwards or downwards along the vertical meridian of the field of view. As the retina was always stationary, the MUA and Δ*V*(*t*) maximum moved over the cortex. Due to the diverse dynamics of the Δ*V*(*t*) when a single moving object enters the field of view, the peak MUA has been considered as the best estimate of the cortical position receiving the retinal signal of the moving bar (Harvey et al., 2009). When the bar moved upwards, the peaks of the MUA and Δ*V*(*t*) moved laterally along the cytoarchitectural border between areas 17 and 18, with a weaker second Δ*V*(*t*) peak moving laterally along the cytoarchitectural border separating areas 19 and 21 (Harvey et al., 2009). When the bar moved downwards, the peaks of the MUA and Δ*V*(*t*) moved medially along these cytoarchitectural borders Movie S1 panel **D**; Figure 2). The representation of the bar in visual areas 17, 18, 19, and 21 always lags the position of the object mapped without delay. This non-delayed representation was on average 50 ms (s.e.m. = 4 ms; *n* = 4) ahead of the maximal Δ*V*(*t*) and the maximal MUA firing rate. See Materials and Methods, Figure A1 and Movie S1 panel **C**. We had no systematic electrode penetrations along the 19/21 cytoarchitectural border.

**Figure 3:**
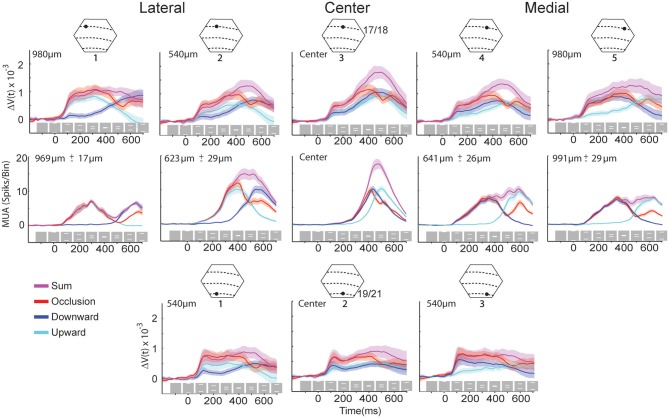
**The Δ*V*(*t*) and MUA at selected spots in the cortex and the sum of the Δ*V*(*t*) and MUA from the single bar conditions**. Above each column is a cartoon of the area imaged by the hexagonal photodiode array where dashed lines indicate the average area borders (see Materials and Methods) and black dots indicate the approximate position of the recording site. The distance from the site representing the center of field of view appears at the top left of each graph. The gray boxes with white bars indicate the position of the stimulus on the monitor for the different time points for the occlusion condition only. Solid lines indicate the mean and shaded regions indicate the standard error of mean, for each trace. For the Δ*V*(*t*) *N* = 14 animals at each site and the MUA represents the average activity across all 16 leads of the laminar probe for *N* = 10 animals at each location.

Thus the motion of a single bar is associated with MUA peaks moving in retinotopic cortical coordinates corresponding to the position of the bar in the field of view (Figure 3).

After the bar appears in the field of view the signal from the VSD increases at cortical sites representing the bars' position and then spreads out in all directions, as has been previously demonstrated (Grinvald et al., 1994; Slovin et al., 2002; Roland et al., 2006; Harvey et al., 2009; Polack and Contreras, 2012). At ~ 120 ms, the neurons in areas 19 and 21 produce a Δ*V*(*t*) increase extending in the direction of motion far ahead of the peak activity mapping the bars' position, (compare Movie S1 panels **D,C)**. This, increase in the population relative membrane potential propagates back to reach the representation of the bar at the 17/18 area border (see Harvey et al., 2009 Figure 5 and Supplementary Movie 7 in that paper). At the 17/18 border a similar slender Δ*V*(*t*) increase is then produced extending on average 8° ahead of the mapping site (Movie S1 panel **D**, 170–205 ms). This slender increase was roughly restricted to the cytoarchitectural border between areas 17 and 18 and hence to the future trajectory of the bar representation. It was therefore referred to as a spatially restricted pre-depolarization, (SRP). Corresponding to the SRP, there was an increase in the MUA recorded from neurons in layers 5, 6, i.e., ahead of those neurons mapping the bar. (see Harvey et al., 2009, Supplementary Movie 7). This MUA increase is more moderate, such that it is easily distinguished from the peak MUA associated with the mapping of the bar. When the neurons spike ahead of those neurons mapping the retinal input, it is like a prediction of the direction the object will move (Figure 2; Movie S1 panel **D**).

### The spatio-temporal dynamics associated with the two bars moving towards each other

In the occlusion condition, the two bars moved toward each other along the vertical meridian, one from above and the other from below the center of the field of view (Figure 1). The spatio-temporal dynamics contained all the characteristics just described for single bar motion, but in double. First two cortical sites of initial increased MUA appeared at the cytoarchitectural border between area 17 and 18 corresponding to the introduction of the bars in the field of view (data not shown). In what follows, we, in accordance with Harvey et al. (2009), use the terms representation and mapping to mean the peak firing in the MUA. Then the Δ*V*(*t*) increased at these two sites and the increase spread laterally. Thereafter two additional sites of Δ*V*(*t*) increases appeared along the cytoarchitectural border between areas 19 and 21, most likely as the result of a feed-forward input from areas 17/18. From 115 ms and onwards, two SRP's extended toward each other along the 19/21 border between the two representations of the bars, (Figure 2, Movie S1 panel **B**). From 115 to 160 ms post stimulus, the two SRP's merged along the 19/21 border. This was followed by an increase of the Δ*V*(*t*)rel, (see Materials and Methods) that propagated from the 19/21 border toward the two representation sites at the 17/18 border, i.e., back propagating synaptic activity (*p* < 0.01, Movie S2). At ~160 ms two similar SRP's appeared extending toward each other in between the moving representations of the bars at the 17/18 area border, (Movie S1 panel **B**, Figure 2).

From 286 ms the MUA increased significantly above the spontaneous rates in the neuron population representing the center of field of view (*p* < 0.01; Table 1, Figure 3).

**Table 1:** **Onsets and peaks of multiunit activity and dye signal**.

		**Medial**		**Central**		**Lateral**
**Recording**	**Site**	**1**	**2**	**3**	**4**	**5**
**MUA 17/18**		**~969 μm**	**~623 μm**	**Center**	**~641 μm**	**~991 μm**
Down	Onset/Peak	525/626	408/536.4	309/436	207/320	177/312
	SEM	7.1/8.9	10.9/9.5	6.1/5.4	11.1/	8.6/9.6
	*N*	7	7	10	5	6
Up	Onset/Peak	180/278	253/396	383/508	467/582	506/633
	SEM	11.9/9.8	14.6/9.9	6.0/4	9.2/8.3	8.42/8.2
	*N*	7	7	10	5	6
Occlusion	Onset/Peak	183/283	226^*^/364^*^	286^*^/413^*^	203/334	187/321
	SEM	11.8/9.1	9.7/6.2	6.42/4.4	10.7/11.5	10.4/11.5
	*N*	7	7	10	5	6
**Δ*V*(*t*) 17/18**		**980 μm**	**540 μm**	**Center**	**540 μm**	**980 μm**
Down	Onset/Peak	270/628	192/556	147/483	124/439	84/349
	SEM	43/20	36/19	28/16	26/25	11.6/34
	*N*	14	14	14	14	14
Up	Onset/Peak	116/332	156/414	164/489	152/528	244/614
	SEM	24/34	34/24	31/21	31/22	50/23
	*N*	14	14	14	14	14
Occlusion	Onset/Peak	106/323	96^*^/364	91^*^/403^*^	97^*^/413	77/336
	SEM	21/38	14/21	14/17	20/37	11.8/43
	*N*	14	14	14	14	14
**Δ*V*(*t*) 17/18**			**540 μm**	**Center**	**540 μm**	
Down	Onset/Peak		193/510	98/431	76/293	
	SEM		43/38	17/48	9/49	
	*N*		14	14	14	
Up	Onset/Peak		122/274	128/412	138/494	
	SEM		37/50	50/50	42/40	
	*N*		14	14	14	
Occlusion	Onset/Peak		62/259	22.2/354^*^	84/250	
	SEM		8/42	12/48	14/40	
	*N*		14	14	14		

* p < 0.01.

Thus the activity during the occlusion condition, while the neuronal populations representing the two bars were still well-separated, displayed all the dynamic characteristics associated with the motion of single bars. That is: moving peak MUA over the cortex, activation of areas 19 and 21 following activation of areas 17/18, SRPs first in areas 19/21 and then after synaptic activity moving from here to areas 17/18 similar SRPs and advanced MUA along the 17/18 border. The only difference was that the SRPs merged and fired neurons at the cortical site representing the center of field of view, i.e., the site of occlusion. This indicates that the brain at this moment could have knowledge about the upcoming clash or occlusion, 126 ms in advance of the occlusion on the display screen (Table 1).

### The approaching phase until occlusion starts

Since the dynamics in visual areas 17, 18, 19, and 21 of the Δ*V*(*t*) (population membrane potential) and MUA up to 370 ms had all the characteristics of the dynamics associated with single bar motion, the dynamics up to 370 ms of the two bars moving toward each other might be a simple combination of single bar dynamics. We therefore tested the hypothesis that the occlusion condition Δ*V*(*t*) and MUA was the simple sum of the Δ*V*(*t*) and MUA in the two control conditions.

We selected five sites along the 17/18 cytoarchitectural border, and hence the path of the moving Δ*V*(*t*) maximum at the 17/18 border, and three sites along the 19/21 cytoarchitectural border for detailed analysis. We calculated the sum of the Δ*V*(*t*) for the control conditions and the sum of the MUA for the control conditions and tested when the amplitude of the Δ*V*(*t*) and MUA for the occlusion condition deviated significantly from these sums. The tests of significance were done using a two tailed *t*-test using a within subjects design. The time courses of the MUA and Δ*V*(*t*) at these sites are shown in Figure 3 for the three motion conditions as well as for the sum of the Δ*V*(*t*) and the sum of MUA of the control conditions.

**Figure 4:**
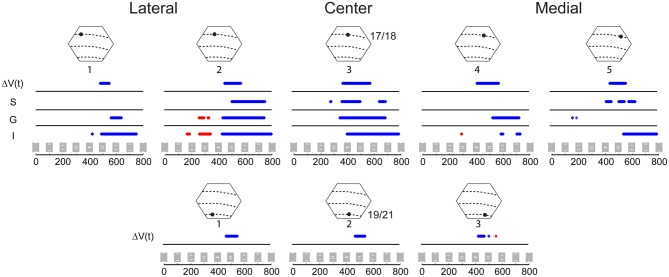
**Statistical comparison of the amplitude of the variables Δ*V*(*t*) and MUA for the occlusion condition compared to the amplitude of the sum of Δ*V*(*t*) and sum of MUA from the single bar conditions**. The MUA was recorded from supragranular (S), granular (G), and infragranular (I) cortical layers at five sites along the 17/18 border **(Top)** and for the Δ*V*(*t*) at three sites along the 19/21 border, **(Bottom)** (Sites identical to those in Figure 3). Epochs where the amplitude of the Δ*V*(*t*) or MUA during the occlusion condition is significantly greater (*p* < 0.01) than the amplitude of the Δ*V*(*t*) sum or the MUA sum in the two single bar conditions are shown in red, and epochs where the amplitude of the Δ*V*(*t*) in the occlusion condition is significantly less (*p* < 0.01) than the amplitude of the sum of the Δ*V*(*t*) or sum of MUA in the single bar conditions are shown in blue.

**Figure 5:**
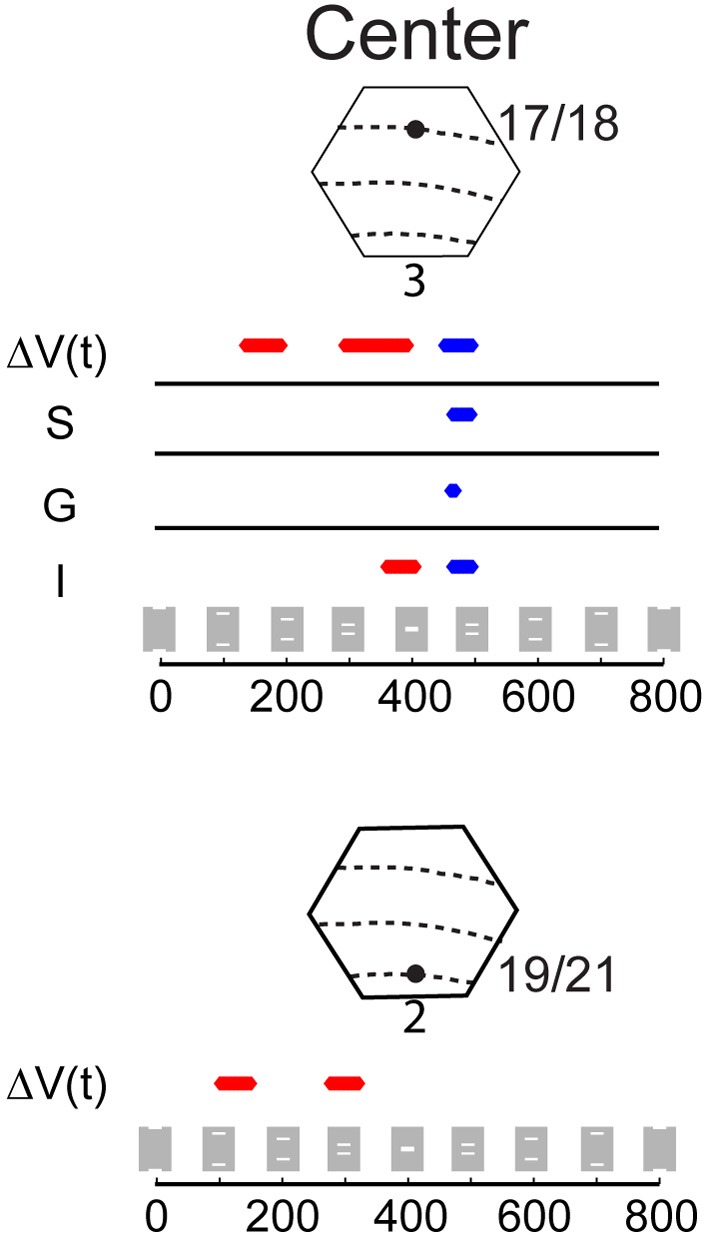
**Statistical comparison of the amplitude of the Δ*V*(*t*) and MUA for the occlusion condition compared to the amplitude of the Δ*V*(*t*) and MUA for the single bar conditions**. The MUA was recorded from supragranular (S), granular (G), and infragranular (I) cortical layers at the cortical site for the center of field of view along the 17/18 border **(Top)** and for the Δ*V*(*t*) at the corresponding cortical site along the 19/21 border **(Bottom)**. Epochs where the amplitude of the Δ*V*(*t*) or the MUA during the occlusion condition is significantly greater (*p* < 0.01) than the amplitude of Δ*V*(*t*) or MUA of either of the two single bar conditions are shown in red, and epochs where the amplitude of Δ*V*(*t*) and MUA in the occlusion condition is significantly less than the amplitude of responding to the single bar conditions are shown in blue (*p* < 0.01 see Materials and Methods). Note that the Δ*V*(*t*) and MUA in the time window 392 to 460 ms was not significantly different from that of a single bar (*p* > 0.2).

Figure 4 shows the results of the statistical tests for the Δ*V*(*t*) as well as for the MUA in the supragranular, granular, and infragranular layers. First, the Δ*V*(*t*) in between the mapping populations of neurons added not significantly different from the sum of Δ*V*(*t*)s in the two control conditions, from 80 ms and onwards to occlusion (392 ms). During most of this time interval, the Δ*V*(*t*) in the occlusion condition was significantly larger than that of either of the single bar conditions. Thus, the linear addition hypothesis could not be refuted. For the rate of the MUA, there were epochs of supra-linear summation during which the firing in the infragranular and granular layers exceeded that associated with the sum of the single bar conditions at a population of neurons 540 μm from the retinotopic point of the center of field of view (Figure 4). Unlike the Δ*V*(*t*), the sum of MUA lateral and medial to the representation of the center of field of view was roughly equal to the MUA of a single bar. This was because the presence of the other bar contributed little to the total MUA at these most lateral and medial positions (Figure 3). In cortex where the center of field of view was represented, however, there was an increase of the MUA in the infragranular layers just prior to occlusion that was significantly larger than that associated with a single bar (Figure 5).

So, whereas one could not refute that the population membrane potentials in the supragranular layers in between the cortical mapping of the two bars added linearly, the MUA was either supra-linear in short epochs or not significantly different from that associated with the similar motion of a single bar, as also seen in Figure 3. In particular, the addition of the Δ*V*(*t*) in supragranular layers did not lead to an increase in the MUA in these layers. It should be noted that the dye signal adds if a larger area of membranes become excited at any measuring point, or, conversely, if already excited dendrites undergo further excitation. Thus, the addition of the Δ*V*(*t*) signal in itself does not imply that the membranes of the dendrites and neurons already excited from one side (say lateral) are identical to those excited from the other side (say medial).

**Figure 6:**
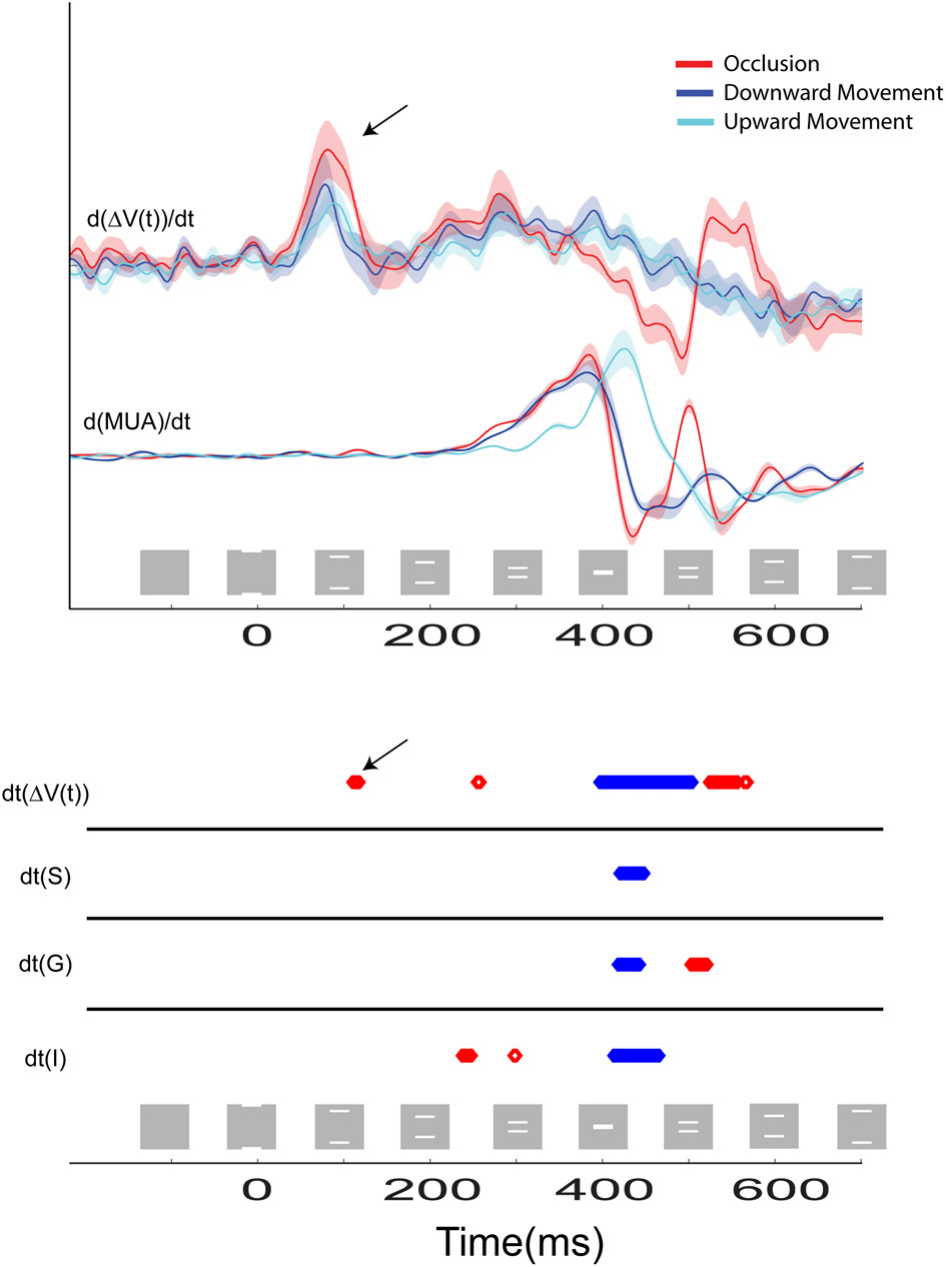
**The time course of the temporal derivatives for the three stimulus conditions are shown**. **Top**: the d[Δ*V*(*t*)]/dt and the d(MUA)/dt. Bold lines indicate the mean values for *N* = 14 and *N* = 10 animals, respectively, and the shaded regions indicate standard error of mean (SEM). Recordings were taken from the cortical site representing the center of field of view at the 17/18 border. The first positive peak at 85 ms is due to the directional pre-excitation ahead of the moving bar representation. **Bottom**: Statistical comparison of the amplitude of the response for the occlusion condition compared to the amplitude of the response for the single bar conditions for the d[Δ*V*(*t*)]/dt and the d(MUA)/dt recorded from supragranular (S), granular (G), and infragranular (I) cortical layers at the site representing the center of field of view. Epochs where the amplitude of these variables during the occlusion condition is significantly greater than the amplitude of the variables to either of the two single bar conditions are shown in red, and epochs where the amplitude of the variables to the occlusion condition is significantly less than the amplitude of responding to either of the single bar conditions are shown in blue (*p* < 0.01). Note that when the first effect of the excitatory synaptic activity reaches the area 17/18 border at 120 ms, the dΔ*V*(*t*)/dt becomes statistically significantly stronger than in the single bar conditions (black arrows **Top** and **Bottom**).

If the Δ*V*(*t*) adds at a given cortical point, the onset latency may diminish compared to the single bar condition. This happened at three measurement points, the point mapping the center of field of view and those two points flanking the center of the field of view at 540 μm (Table 1). Also the onset of MUA occurred earlier at the cortical site representing the center of field of view (Table 1). At this site, both the onset (286 ms) and peak time of the MUA came equally earlier in the occlusion condition than in the control conditions by 23 ms (see Materials and Methods for judgment of latencies). The onset and peak of the Δ*V*(*t*) recorded from this site at the 17/18 border were also advanced, in this case by 73 and 80 ms, respectively. At the site mapping the center of field of view at the 19/21 border, the timing of the peak of the Δ*V*(*t*) arrived an average of 113 ms earlier than for the control conditions in a pair-wise comparison in which the animal was its own control (Table 1). Thus, as the populations of neurons firing to the moving bars came within 600 μm of one another, the MUA and Δ*V*(*t*) peaked earlier than in the control conditions. At the center of the field of view the derivative of the VSD signal, d[Δ*V*(*t*)]/dt, also became significantly greater than that that for single bar conditions, and this occurred already at 85 ms post stimulus, (Figure 6 arrows; Movie S3).

### Occlusion dynamics

Eventually, the two peaks of MUA, representing the two moving bars, moved closer to the cortical point representing the center of field of view. At 392 ms the bars abutted one another on the display screen and the occlusion became maximal at 412 ms (Figure 1, Movie S1). At 370 ms the dΔ*V*(*t*)/dt started to decrease at the cortical site representing the center of field of view (Figure 6). At this point in time the bars' distance on the screen was 2.6°, and the bar representations at the 17/18 border in retinotopic coordinates were further apart, given the retino-cortical delay.

*In vivo*, the derivative of Δ*V*(*t*), the d[Δ*V*(*t*)]/dt is an indicator of net membrane excitation and net inhibition (Ferezou et al., 2006, 2007; Berger et al., 2007; Eriksson et al., 2008; Roland, 2010). If the d[Δ*V*(*t*)]/dt increases significantly over the pre-stimulation baseline this indicates net membrane excitation, if the d[Δ*V*(*t*)]/dt decreases significantly below baseline, this indicates reduction of net-excitation and most likely increase of net inhibition. The d[Δ*V*(*t*)]/dt of the neurons mapping the center of field of view at the 17/18 border (and identical to those mapping the occlusion) started to decrease at 370 ms (Figure 6). Thereafter the Δ*V*(*t*) peaked at 403 ms. The MUA peaked at 413 ms when the occlusion on the screen was maximal. The MUA in the supragranular, granular and infragranular layers at this cortical spot and at this moment scaled to that of a single bar (Figure 5), as did the total MUA (Figure 3). In the supragranular, granular and infragranular layers, the MUA was not statistically different from the MUA associated with a single bar from 415 to 460 ms (*p* > 0.2, Figure 5).

At the cortical point mapping the center of field of view, the d[Δ*V*(*t*)]/dt, then the d(MUA)/dt and subsequently the MUA in all layers continued to decrease, such that the d[Δ*V*(*t*)]/dt, Δ*V*(*t*) and the MUA were significantly below the values associated with a single bar at 460–500 ms (*p* < 0.005) (Figures 5, 6). This significant dip in the d[Δ*V*(*t*)]/dt, far below the baseline, may be interpreted as a net inhibition of the population. Accordingly the MUA also diminished significantly following the d[Δ*V*(*t*)]/dt decrease. This raised the question of what might have caused this. We therefore looked at the d[Δ*V*(*t*)]/dt over all neurons in the supragranular layers of areas 17, 18, 19, and 21. From 370 ms there was a strong decrease in d[Δ*V*(*t*)]/dt starting at the four zones of cortex where the bars were mapped initially (*p* < 0.025, Figure 6). The significant d[Δ*V*(*t*)]/dt decrease propagated from these zones following the subsequent cortical trajectory across the neuron populations subsequently mapping the bars. At 473 ms the d[Δ*V*(*t*)]/dt decrease reached its minimum almost simultaneously over all four areas. The decrease though remained the strongest at the cortical bar trajectory zones (Movie S3). The Δ*V*(*t*) also decreased, because of the strong d[Δ*V*(*t*)]/dt decrease (Figure 3).

Although net-inhibition appeared first in the populations of neurons that had already mapped the approaching bars, this cannot explain why the population of neurons in the supragranular layers at the cortical spot mapping the center of field of view decreased *prior* to the time this population actually mapped the bars and their subsequent occlusion. It is likely therefore that there might be more than one mechanism reducing the d[Δ*V*(*t*)]/dt.

### The population membrane and MUA dynamics when the two bars moved away from each other after the occlusion

After the occlusion was maximal on the screen at 412 ms, the bars at the display screen formed one growing rectangle until 435 ms, at which point they began to move away from one another in opposite directions.

As seen in Figure 6, the d(MUA)/dt started to increase significantly from 500 ms, first in the granular layer. Thereby the MUA and subsequently the d[Δ*V*(*t*)]/dt also increased at the cortical spot mapping the center of field of view. The d[Δ*V*(*t*)]/dt increase appeared also outside the bar representations in areas 17, 18, 19, and 21 in the time interval from 473 to 515 ms (Movie S3). As is also apparent from Figure 6, these increases in d(MUA)/dt and d[Δ*V*(*t*)]/dt were transient, significant (*p* < 0.01), and unique to the occlusion condition (see also Figure 3). The MUA increase was especially strong in the granular layer (Figure 6) and followed by an increase of d[Δ*V*(*t*)]/dt (Figure 6). This resembles the dynamics seen after excitation by thalamo-cortical afferents (Roland et al., 2006; Harvey et al., 2009). The increase of MUA at 525 ms, however, occurred only at the cortical spot mapping the bar occlusion (Figure 3).

Perhaps the most conspicuous finding was that the MUA never recovered fully at their subsequent trajectory across the populations of neurons that once already had mapped the bars. This decrease in MUA compared to the single bar MUA was strongly significant for the MUA across layers at the positions outside the retinotopic spot of the center of field of view (*p* < 0.01, Figure 3). In the control conditions, the populations of neurons mapping the single bar also significantly decreased their d[Δ*V*(*t*)]/dt, resulting in a decrease in Δ*V*(*t*) some 200 ms after the peak MUA and peak Δ*V*(*t*) as seen in Figure 2. These decreases in d[Δ*V*(*t*)]/dt and Δ*V*(*t*) are relatively broad.

In summary, when two bars move toward each other along the vertical meridian in the field of view and the retinas are still, the bars are mapped by two continuously moving maximal laminar MUA increases moving toward each other in constantly changing populations of neurons located at the border between areas 17 and 18. Peak Δ*V*(*t*) activity also moved toward each other over populations of neurons along the border between areas 19 and 21 and toward each other along the border between areas 17 and 18. Further, the population of neurons along the 19/21 border generated net excitatory (synaptic) membrane activity propagating toward the area 17/18 border where SRP's appeared in the population of neurons located in between the moving peak MUA and peak Δ*V*(*t*). Almost until the occlusion, these SRP's were double the amplitude of those generated by single bar motion. The neurons between the moving peaks in the MUA then started to fire, especially in the infragranular layers. The neurons representing the center of the field of view then fired, 126 ms prior to the occlusion. When the neuronal populations representing the bars in areas 17/18 were ~600 μm apart, the activity in the population of neurons between them showed strong decreases in d[Δ*V*(*t*)]/dt, and subsequently the MUA and Δ*V*(*t*). At the time of occlusion the MUA and Δ*V*(*t*) matched those of a single bar. The d[Δ*V*(*t*)]/dt behind the moving mapping populations was now also strongly decreased. After a short transient increase in MUA and then in Δ*V*(*t*) at the cortical site representing the center of the field of view, the Δ*V*(*t*) recovered somewhat, but the MUA remained significantly reduced in the populations of neurons where the bars were mapped following occlusion.

## Discussion

Whereas there is a rich psychophysical literature showing interactions between continuously moving objects, the neurophysiologic mechanisms of these interactions have not previously been examined. We examined two bars moving toward each other and then occluding one another at the center of field of view. There are at least five types of interactions between the populations of neurons in areas 17, 18, 19, and 21 relevant for the understanding of the observed dynamics: (1) feed-forward passage of action potentials between layers and areas, (2) synaptic activity from higher to lower areas, (3) interactions between the populations mapping the bars, (4) interactions between the populations of neurons representing the bars and the population of neurons in between them, and (5) net inhibitory effects from the population that once mapped one of the bars and subsequently mapped the other bar.

We examined only occlusion taking place in the center of field of view. Consequently we cannot generalize our findings to dynamic object occlusions elsewhere. Also the bars had identical contrasts, such that their leading edges were no longer apparent from the moment the occlusion started. Rather they appeared to merge and shrink to one single bar at the moment of maximal occlusion. Moreover, we had no systematic electrode penetrations along the cortical path of motion in areas 19 and 21 and cannot therefore with certainty state the position of the bar representations here.

### Inter-area feed-forward and back propagating activity

Initially, just after the bars appeared, there were no differences between the single bar and the occlusion condition. Subsequently, the bars were represented, presumably through feed-forward communications, to two populations at the area 19/21 border. This was verified in the few examples where we had the appropriate electrode penetrations [data not shown, but see also Harvey et al. (2009), Roland (2010)]. The feed-forward flow of action potentials from the lateral geniculate nucleus to areas 17/18 continued, with modulations, throughout the time course of bar motion, continuously moving the two MUA peaks closer to occlusion. After occlusion, feed-forward excitation from the lateral geniculate nucleus moved the peaks of the MUA away from each other.

After the neurons along the border between areas 19 and 21 had produced SRP's, the neurons of area 18 and 19 showed increases in Δ*V*(*t*) and d[Δ*V*(*t*)]/dt as an organized wave from 115 ms to 160 ms (Movie S2). As the Δ*V*(*t*) signal reflects differences in synaptic activity (Berger et al., 2007) and the d[Δ*V*(*t*)]/dt increase reflects net increase in membrane excitation, one could interpret this wave as a propagation of synaptic excitatory activity from the area 19/21 border toward the 17/18 border. Movie S3 shows an 11 ms delay in d[Δ*V*(*t*)]/dt increase between the mapping site at the 19/21 border and that of the 17/18 border 60–71 ms after stimulus onset. This could arise from other causes than transmission of action potentials between these areas. However, in Movie S2 one can follow the propagation over cortex of the relative Δ*V*(*t*) peak with fast velocity from 19/21 to 17/18. The cortical motion of this peak also includes retinotopic positions in areas 19 and 18 that are not supposed to be stimulated by the stimulus. This, however, is a characteristic of these (waves of) back propagating synaptic excitation (Roland et al., 2006; Ahmed et al., 2008; Harvey et al., 2009). They seem to have a course similar to the course of the feedback axons in the ferret (Cantone et al., 2005).

When the synaptic activity reached the 17/18-area border, it added to the net-excitation of the sub-population of neurons in between the populations of neurons representing the moving bars. This might have contributed to bring some of these neurons over their firing threshold. Harvey et al. (2009) measured a similar propagation of d[Δ*V*(*t*)]/dt in the same time interval, elicited by the motion of a single bar. Such motion of (net excitatory) synaptic activity is also observed in other species and other visual stimulus conditions (Eriksson and Roland, 2006; Roland et al., 2006; Xu et al., 2007; Ahmed et al., 2008; Takagaki et al., 2008; Harvey et al., 2009; Roland, 2010; Ayzenshtat et al., 2010).

### Intra-area interactions

Interactions were observed in all four areas beginning 85 ms post stimulus. These interactions began in the population of neurons in between the moving representations of the bars. During the occlusion condition the Δ*V*(*t*) in the supragranular layers was not significantly different from the sum of the single bar conditions. We cannot discern how much of this summation was due to the recruitment of independent, for example directionally tuned, neurons, or to the increased drive on neurons responding to both directions of bar motion. Since the MUA started to increase significantly earlier in the infragranular layers, this indicates that at least some neurons in these layers could be influenced by additive net excitations. Also in the infragranular layers, there were non-linearly additive epochs of MUA. This was despite the fact that the moving bars were not collinear (Chisum et al., 2003), but in accordance with reports of firing ahead of the object mappings, (Guo et al., 2007; Harvey et al., 2009).

The synaptic net excitation between the moving representations could be mediated by horizontal connections extending from the bar representations in the lower supragranular layers. The reason why the Δ*V*(*t*) sums along the future path in between the moving bar representations could be that the populations of neurons representing the bars, through excitatory horizontal connections (Bosking et al., 1997; Chisum et al., 2003; Buzas et al., 2006), increased the synaptic net excitation along the future path of the bars' motion. In addition action potentials from higher order areas 19/21, where the future path was already mapped, could further increase the Δ*V*(*t*) along the future path in area 17/18, (Harvey et al., 2009).

After 180 ms the neurons, especially in the infragranular layers, started to fire in between the moving bar representations (Figure 3 and Table 1). In the occlusion condition the premature firing also reached the cortical zone for the future occlusion (280 ms) indicating that the brain at this point had information to predict the occlusion. As the firing was strongest in infragranular neurons and as the vast majority of neurons in primary visual cortex projecting to superior colliculus are in layer 5, (Palmer and Rosenquist, 1974), one may speculate that this premature firing toward the cortical point of future occlusion could be useful for generating a saccade to the point in the field of view where the occlusion was expected in analogy with parietal cortical neurons (Duhamel et al., 1992).

### Inhibition prior to occlusion

The d[Δ*V*(*t*)]/dt is related to the inward/outward currents of the cells in the upper layers of cortex. This follows from the near linear relation between the population membrane potentials in supragranular layers and the Δ*V*(*t*), (Petersen et al., 2003; Ferezou et al., 2006; Berger et al., 2007; Eriksson et al., 2008; Roland, 2010). When the distance between the moving bar representations at the 17/18 border were approximately 600 μm and 20 ms prior to the start of the occlusion of the bars on the display screen, the d[Δ*V*(*t*)]/dt went below baseline and continued to decrease. After the d[Δ*V*(*t*)]/dt went below baseline, the dMUA/dt in all layers decreased almost simultaneously. Together this indicates a decrease in excitation, or alternatively an increase of inhibition of all layers or both, at the central position of the 17/18-area border. What is in favor of an increased net inhibition is that the d[Δ*V*(*t*)]/dt went far below baseline and that the dMUA/dt followed this decrease (Figure 6). From the measurements depicted in Movie S3 one can see that the d[Δ*V*(*t*)]/dt decreased all along the path taken by the bar representations until occlusion. The spatial dynamics of d[Δ*V*(*t*)]/dt in the interval 370–570 ms is complex. For this reason first the net inhibition of the population of neurons that subsequently mapped the occlusion is discussed.

The inhibition of the population of neurons mapping the occlusion/the center of field of view could depend on several mechanisms. When the bars on the screen came closer together, the likelihood increases that neurons located close to the cortical point representing the center of field of view where the occlusion is going to take place may react. One mechanism could be that the geniculo-striate afferents exciting the granular layer neurons also contact basket cells providing almost simultaneous inhibition (Ahmed et al., 1994; Liu et al., 2011). If such elicited extra inhibition reaches the upper layers, the d[Δ*V*(*t*)]/dt might decrease. Contradicting the geniculo-striate mechanism of feed-forward inhibition, is that the decrease started in supragranular layers prior to the time when the d(MUA)/dt decreased. Another possibility is that the lateral geniculate neurons might have been inhibited. However, the lateral geniculate neurons cannot have been very much inhibited, as the MUA in layer 4 of the cortex was, at the time of occlusion, equal to that of a single bar.

Another alternative is that the increased inhibition of the population of neurons mapping the center of field of view is elicited intra-cortically by the horizontal connections.

The majority of the neurons in area 17 decreases their firing rates to counter-phase gratings and oppositely moving bars (Baker and Emerson, 1983; Qian and Andersen, 1995). *In vitro* experiments on the ferret visual cortex supragranular layers also show that simultaneous stimulation of cortical points separated by 500 μm or less generates net inhibition in neurons getting synaptic excitation from both stimulating points in these layers in between the stimulation sites (Tucker and Katz, 2003). If the inhibition was elicited by contrast edges approaching each other, theoretically the inhibition should cease when the cortex detected that occlusion was maximal. The contrast edges, both the leading and the following edges of the bars, then would move away from the cortical site of the center of field of view. This was what happened. One may accordingly describe the behavior of the neurons, at and close nearby the central field of view representation at the 17/18 border, as being net inhibited by the simultaneous and oppositely moving excitation associated with the bar representations. This mechanism might require increased firing of local inhibitory neurons in between the bar representations. The horizontal connectivity in area 17 is most pronounced in lower layer 3 and layer 5 (Gilbert and Wiesel, 1989; Buzas et al., 2006). The net inhibition in these layers may have helped in bringing the MUA in phase across all layers when the granular layer mapped just one bar at the midst of the occlusion (Figures 3, 5). According to Figure 6, the net inhibition or depression of the firing ceased first in the granular layer at 462 ms, i.e., 50 ms after the maximal occlusion on the screen.

### The reduction of the multiunit activity after the occlusion

As seen in Figure 5, the MUA at the site mapping the center of field of view was for a short period below that associated with a single bar just after the occlusion. As the MUA started to increase in the granular layer, the increase spread to supra and infragranular layers and increased the dΔ*V*(*t*)/dt to a temporary maximum in about 50 ms, which is the time it normally takes to increase the population dΔ*V*(*t*)/dt when a stimulus appears (Harvey et al., 2009; Roland, 2010).

Although the inhibition at the cortical retinotopic site of the center of field of view may be explained by mutual horizontal inhibition in areas 17/18 from the bar edges moving toward each other, this cannot explain why the Δ*V*(*t*) decreased *behind* the moving bar representations (Movies S1, S3, Figure 4). Neither can this explain why the populations of neurons representing the vertical meridian uniformly suppressed the MUA, after the occlusion. One major result was that the total MUA, across layers, after the occlusion, was significantly reduced at all cortical points where the bars had been mapped prior to the occlusion. As seen in Figure 3, and in Harvey et al. (2009), there is no reduction of the total MUA when single bars get mapped. The total MUA associated with single bar representation moving over cortex thus is symmetrical, no matter whether the motion is toward or away from the center of field of view. However, when the representation of a single bar moves over the cortex, the dΔ*V*(*t*)/dt of the neurons mapping the moving bar turns negative with a delay of 130–150 ms (Roland, 2010). This significant negativity thus is a sign of net inhibition.

As seen in Figure 3, this reduction of MUA is relatively long lasting. One possibility is that the cortex remains in a generally inhibited state after the inhibition associated with the bars approaching occlusion. This is unlikely for several reasons. First, the decrease in d[Δ*V*(*t*)]/dt started behind the mapping populations prior to the occlusion. Second, the inhibition was released in the population of neurons representing the occlusion, increasing the MUA to the level of a single bar (Figure 3). Third, the d[Δ*V*(*t*)]/dt did not remain suppressed, but showed a clear rebound above that associated with single bars and stayed normal. However, at 570 ms, i.e., 150 ms after the mapping of the occlusion at the d[Δ*V*(*t*)]/dt started to decrease again, but only in the zone mapping the center of field of view from where it spread slowly (Movie S3).

The mapping of single bars, however, is also associated with a significant decrease in d[Δ*V*(*t*)]/dt below baseline after some 100–150 ms (Roland, 2010). This affects also the Δ*V*(*t*) that eventually becomes negative (Figure 2). One possibility is that the mapping of the bar is associated with a delayed inhibition (afterhyperpolarization?) that lasts longer. In the occlusion condition, this delayed inhibition then reduces the MUA when the retinal input reaches the population that mapped the other bar 150 ms ago or earlier.

## Conclusions

The spatio-temporal dynamics of membrane potential changes and laminar MUA associated with objects moving toward occlusion and continuing thereafter is complex. When the retina is still, a single bar moving in the field of view is mapped retinotopically as peak increases in firing rates across cortical layers and, after some 150 ms also by peak increases in membrane potentials by a populations of neurons in each of the four visual areas 17, 18, 19, and 21. If the bar moves up or down the vertical meridian, the laminar peak increases follow paths over the cortex that correspond to the retinotopic mapping of events located at the vertical meridian. In cortex one path is equal to the cytoarchitectural border between areas 17 and 18 and another path equal to the cytoarchitectural border between areas 19 and 21 and yet other paths at several locations in other areas that were not explored in our study. The neurons that map the object at each position in the field of view in each area form a path over the cortex corresponding to the trajectory in the field of view, the path population.

When two objects move exactly toward each other, the path population is identical for the two objects. The instantaneous mapping of the moving objects was done by the laminar peak firing of two constantly approaching sub-populations of neurons at the 17/18 border. At the border between areas 19 and 21 two net excitations of the population membrane potentials appeared approximately 50 ms after stimulus onset. This excitation presumably derives from feed-forward connections emanating from the neurons representing the bars at the 17/18 border. Early on, the neurons of the path population at the 19/21 border in the sector between the peak net membrane excitation also became net-excited. The second interaction between the path populations was a back propagation of net excitatory synaptic activity 115–160 ms after the start of motion from the 19/21 path population to the 17/18 path population.

The interactions expressed in the path population of neurons, in the sector between the neurons mapping the progression of the bars, started 85 ms post stimulus onset with the d[Δ*V*(*t*)]/dt, net membrane excitation, propagating to the populations mapping the center of field of view from both sides. This continued with the formation of net membrane excitation and increased membrane potential of double the amplitude of both of the whole path population of neurons (17/18 and 19/21) in the upper layers. This behavior of the Δ*V*(*t*) thus could be interpreted as a long range horizontal interaction combined with the effects of an excitatory back transmission from areas 19 and 21.

Despite the additive effect of the relative population membrane potentials in the supragranular layers of the 17/18 population, the MUA in these layers did not deviate from that associated with a single bar, suggesting that the Δ*V*(*t*) effect was mainly subthreshold. However, in the infragranular layers, the MUA started earlier and far ahead of the peak activity mapping the bars. Already at 286 ms when the bars were 7° apart, the significant firing in infragranular layers reached the cortical zone mapping the future site of the occlusion. This indicates that the brain from this moment had information to predict a collision or an occlusion. This finding and the following findings were particular to the occlusion condition.

When the laminar MUA associated with the moving bars came closer, the dΔ*V*(*t*)/dt turned negative and thereafter the MUA decreased simultaneously in all layers. This we interpret as a net inhibition of the membranes in the cortical zone of the occlusion. So far these interactions in the 17/18 path population between the mapping populations may be described as horizontal interactions. At the time of occlusion in the cortex at 413 ms (Table 1) the spiking population was one population of neurons spiking with a peak rate corresponding to that of a single bar. After the occlusion the sub-populations mapping the bars moving away from each other became identical to the neurons that had once already mapped the bar moving in the opposite direction. Despite a short rebound starting with firing in the granular layers, the MUA of the bar mapping populations did not recover to that prior to the occlusion. We attribute this relatively long lasting depression of the spiking occurring with a delay of 130–150 ms to a delayed inhibition/after hyperpolarization.

The feed-forward and back transmission (feedback) interactions between the path populations of neurons of different areas and the local (horizontal) excitatory interaction between the mapping subpopulation and the sector of the path population ahead occur in association with movement of single bars as well as two bars moving to occlusion. The local net-inhibitory interactions at short range, and the delayed and long lasting inhibition of the spiking of the mapping neurons when the neurons that once already mapped the approaching bars now again must map the departing bars, are specific for the occlusion condition in these experiments. It remains to be investigated whether the local short-range inhibition is a general phenomenon for any two bars approaching each other, no matter at which angle.

### Conflict of interest statement

The authors declare that the research was conducted in the absence of any commercial or financial relationships that could be construed as a potential conflict of interest.

## Supplementary material

The Supplementary Movies for this article can be found online at: http://www.frontiersin.org/Systems_Neuroscience/10.3389/fnsys.2013.00023/abstract

Movie S1 **The Δ*V*(*t*) of the occlusion condition and the estimate of the position of the cortical position of the representations of the bar without delay in one animal. (A)** Animation of the stimuli in the occlusion condition with a frame frequency of 200 Hz. Distance from the center of field of view shown on the sides of the monitor. Time in ms from the start of motion is shown in green. **(B)** The voltage sensitive dye signal, Δ*V*(*t*), in the occlusion condition. The scale to the right shows the absolute values. Yellow and colors above yellow signify significant changes (*p* < 0.01). Notice the two spatially restricted depolarizations starting along the 19/21 border (115 ms), and then shortly after along the 17/18 border. For location of cytoarchitectural borders in this animal see C and D. Notice also the almost simultaneous decrease in the Δ*V*(*t*) starting at 445 ms. **(C)** Location of the bar representation without delay in response to single bars, here shown as downward moving, after (Kalatsky and Stryker, 2003) (see details in Figure A1). **(D)** Bonferoni corrected (*p* < 0.01) and normalized, Δ*V*(*t*)rel(s), signal for a downward moving bar. This movie shows only the significant responding after the presentation of a bar moving downward along the vertical meridian.

Movie S2 **The increased net membrane excitation traveling from areas 19/21 toward areas 17 and 18. (A)** Animation of the stimuli in the occlusion condition with a frame frequency of 200 Hz. Distance from the center of field of view shown on the sides of the monitor. Time in ms from the start of motion is shown in green. **(B)** The statistically significant part of Δ*V*(*t*)rel (phase plot) (*p* < 0.01) averaged across animals in response to two bars moving to occlusion. The feedback began at 120 ms after stimulus onset and traveled toward the 17/18 border with an average velocity of 0.12 mm ms^−1^ where it arrived at 160 ms (standard error of mean 10 ms, *n* = 14). The signal then decreases as the bars approach one another. This decrease then surrounds the site mapping the occlusion (representing center of field of view), black circle.

Movie S3 **The spatio-temporal increases, back-propagation from areas 21 and 19, and subsequent decrease of d[Δ*V*(*t*)]/dt averaged over three animals**. Statistically significant increases are shown yellow and red; statistically significant decreases are shown light blue and blue [*p* < 0.025 false discovery rate with correction for multiple comparisons (Materials and Methods)]. The cytoarchitectural borders are aligned (Methods) across animals. Notice also the net inhibition of the membrane potentials starting bilaterally in the periphery. The scale values shown in the scale bar for the d[Δ*V*(*t*)]/dt should be multiplied with 10^−6^.

## Abbreviations

MUA: multiunit activity
VSD: voltage sensitive dye
SRP: spatially restricted pre-depolarization.
